# Interspecific Drought Cuing in Plants

**DOI:** 10.3390/plants12051200

**Published:** 2023-03-06

**Authors:** Omer Falik, Ariel Novoplansky

**Affiliations:** 1 Achva Academic College, Arugot 7980400, Israel; 2 Mitrani Department of Desert Ecology, Blaustein Institutes for Desert Research, Ben-Gurion University of the Negev, Sede Boqer Campus, Midreshet Ben-Gurion, 8499000, Israel

**Keywords:** *Cynodon dactylon*, drought stress, phenotypic plasticity, interspecific plant communication, root communication, *Stenotaphrum secundatum*, stomata, stress cues

## Abstract

Plants readily communicate with their pollinators, herbivores, symbionts, and the predators and pathogens of their herbivores. We previously demonstrated that plants could exchange, relay, and adaptively utilize drought cues from their conspecific neighbors. Here, we studied the hypothesis that plants can exchange drought cues with their interspecific neighbors. Triplets of various combinations of split-root *Stenotaphrum secundatum* and *Cynodon dactylon* plants were planted in rows of four pots. One root of the first plant was subjected to drought while its other root shared its pot with one of the roots of an unstressed target neighbor, which, in turn, shared its other pot with an additional unstressed target neighbor. Drought cuing and relayed cuing were observed in all intra- and interspecific neighbor combinations, but its strength depended on plant identity and position. Although both species initiated similar stomatal closure in both immediate and relayed intraspecific neighbors, interspecific cuing between stressed plants and their immediate unstressed neighbors depended on neighbor identity. Combined with previous findings, the results suggest that stress cuing and relay cuing could affect the magnitude and fate of interspecific interactions, and the ability of whole communities to endure abiotic stresses. The findings call for further investigation into the mechanisms and ecological implications of interplant stress cuing at the population and community levels.

## 1. Introduction

Coping with environmental variation is one of the most prominent and ubiquitous challenges of biological existence. At the population level, it is one of the major drivers of Darwinian evolution and genetic diversity [1]. However, at spatiotemporal scales relevant to individual organisms, behavioral responses and phenotypic plasticity have clear adaptive advantages [2,3,4,5,6,7,8,9,10]. In contrast to natural selection that does not require organismal awareness or involvement, adaptive behavior and phenotypic plasticity are based on the ability of individuals to perceive and integrate accurate information relevant to challenges and opportunities in their immediate environments [11,12]. Unlike rapid biochemical, physiological, and some behavioral responses, developmental plasticity could require substantial time, and, thus, relevant information must pertain to anticipated rather than to prevalent conditions. Accordingly, given sufficiently tight corrections between predictive cues and signals, and ensuing conditions, natural selection is expected to favor preemptive responses to forthcoming rather than to current conditions [8,13,14,15,16]. Highly relevant and reliable information is often available from conspecific neighbors that already experience environmental changes and challenges, such as in the case of bacterial quorum sensing [17] or interplant communication of warning cues related to herbivory (e.g., refs. [18,19,20]), salinity [21], or pathogen attack [22,23,24].

Useful information could also be perceived from other species. For example, many animals readily respond to allospecific alarm calls related to the presence of predators [25,26,27,28], (but see [29]). As most plants require the same resources and are susceptible to similar stresses and enemies, it is not surprising that many plants are able to take advantage of cues emitted from taxonomically remote neighbors. Besides their ability to communicate with their pollinators, herbivores, predators, and pathogens of their herbivores, and myriad symbionts, many plants can emit, eavesdrop on, and respond to a large variety of cues released from plants belonging to other species [30]. In one of the most studied systems, wild *Nicotiana attenuate* plants have been demonstrated to incur significantly lower herbivore damage when receiving volatile cues from neighboring damaged *Artemisia tridentata* shrubs [31]. Similarly, UV-C-stressed *Arabidopsis thaliana* and *Nicotiana tabacum* plants readily exchange volatile cues with their neighbors regardless of their taxonomic identity [32]. Interestingly, in some cases, interspecific communication is facilitated by mycorrhizal networks, indicating that environmental information can be readily transmitted and relayed across kingdom barriers, e.g., refs. [33,34].

We previously demonstrated that unstressed *Pisum sativum* plants rapidly close their stomata in response to interplant cuing from drought-stressed conspecific neighbors, and that ‘relay cuing’ can elicit stomatal closure in multiple increasingly distant unstressed plants [35]. Interplant drought cuing and relay cuing were only observed between plants that shared their rooting media, implying reliance on root–root communication rather than on aboveground volatile cuing [35]. The involvement of ABA in interplant drought cuing has been demonstrated from experiments in which interplant drought cuing was drastically reduced in plants with diminished ABA synthesis [16] and additional analyses showing elevated ABA levels in the rhizosphere of both drought-stressed plants and their unstressed neighbors [36].

An additional study demonstrated both direct and relayed interplant drought cuing in the wild plants *Cynodon dactylon*, *Digitaria sanguinalis,* and *Stenotaphrum secundatum* [37]. In a recent study, we have shown that cuing from drought-stressed plants significantly increased the survival of both directly and relayed-cued target plants under drought [16].

Here, we tested the hypotheses that plants are able to perceive and respond to both direct and relayed interspecific drought cuing and that responsiveness to drought cuing relies on the identity of the emitting plant and its inherent drought tolerance. Responsiveness to drought cuing was studied by following stomatal aperture in unstressed relatively xeric *C. dactylon* and relatively mesic *S. secundatum* plants that were subjected to either direct or relayed drought cues from intra- or interspecific neighbors.

## 2. Results

### 2.1. Intraspecific Drought Cuing

**Figure 1:**
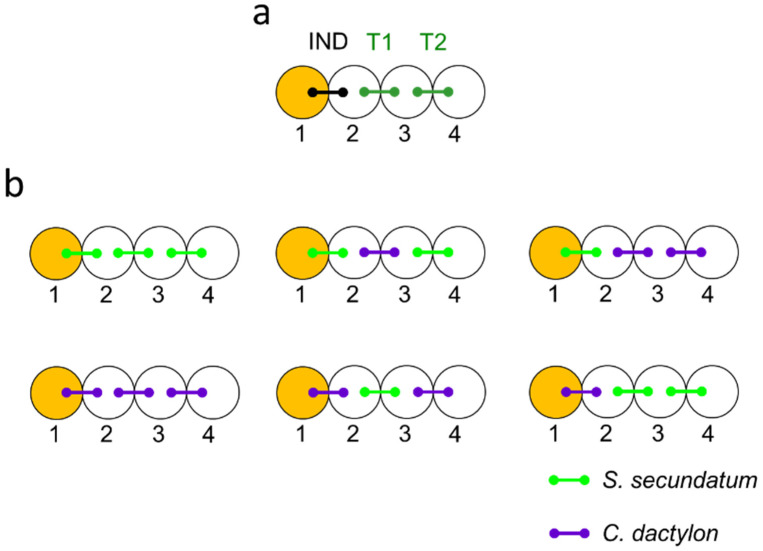
Testing for interspecific drought cuing—the experimental setup. Circles represent pots and connector lines represent split-root plants. Plants neighboring an externally stressed plant (IND) shared their pots with their immediate unstressed (T1) neighbors and target plants either shared their other pots with another target plant (T1) or only with their immediate neighbors (T2) (**a**). Drought or control treatments were imposed by replacing the water in pot 1 (orange) with either dry (drought) or wet (benign control) vermiculite–bentonite (VB) mixture for 60 min. Stomatal width was measured in paired triplet sets. interplant drought cuing was tested and compared between treatments in which the identity of the IND and target plants varied (**b**), to reveal the ability of both species to emit and respond to both direct (T1) and relayed (T2) drought cues from either intra- and interspecific neighbors.

**Figure 2:**
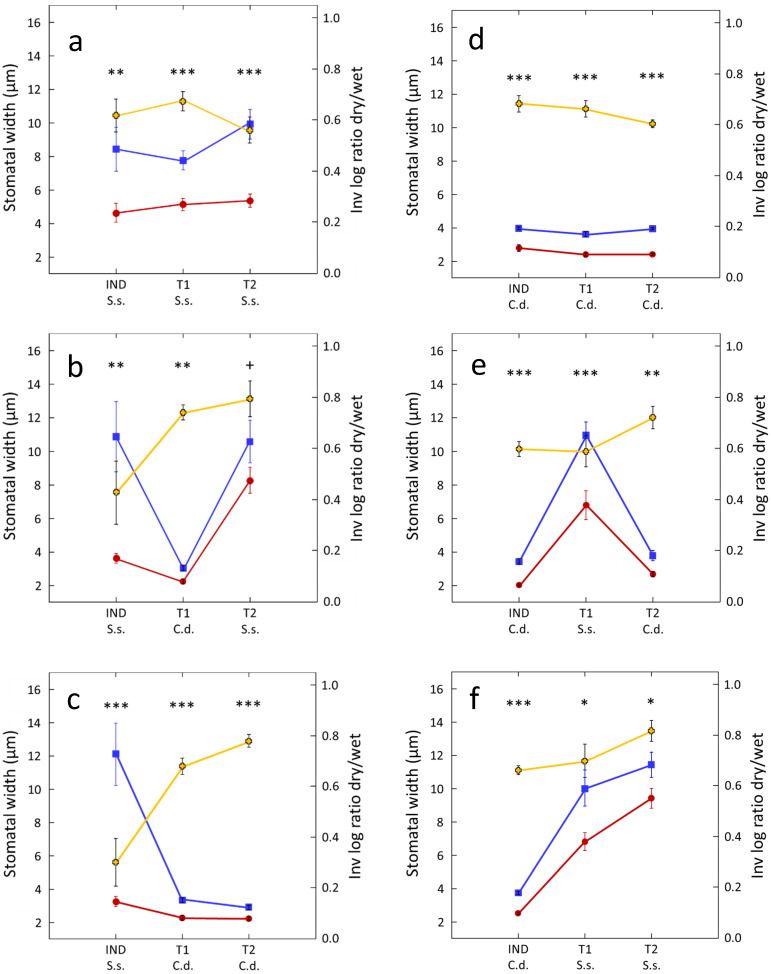
Testing for interplant drought cuing in *S. secundatum* (S.s.) and *C. dactylon* (C.d.). Data (mean ± 1 SEM; *n* = 12), are for stomatal width of treated IND plants and their untreated (T1, T2) target neighbors in benign control triplets (blue lines) and drought-cuing triplets (red lines). IND-T1-T2 triplets comprised different combinations of S.s and C.d.: S.s-S.s-S.s (**a**); S.s-C.d.-S.s (**b**); S.s-C.d.-C.d. (**c**); C.d.-C.d.-C.d. (**d**); C.d-S.s-C.d. (**e**); C.d-S.s.-S.s. (**f**). To properly compare the relative effects of drought (IND plants), drought cuing (T1 plants), and relayed cuing (T2 plants) on stomatal aperture regardless of absolute stomatal width, a second y-axis (right) presents the inverse of the log of (treated/control) ratio of each data pair (yellow lines). Paired *t*-test, + 0.1 < *p* > 0.05; * *p* < 0.05; ** *p* < 0.01; *** *p* < 0.001.

As expected, both *S. secundatum* and *C. dactylon* demonstrate interplant drought cuing and relayed cuing. Subjecting one of the roots of the IND plant to 60 min of drought (pot 1, Figure 1) causes 28–45% and 30–39% decreases in stomatal aperture, in drought-treated or cued *S. secundatum*, and *C. dactylon* triplets*,* respectively, compared to their unstressed controls (Figure 2a,d). Stomatal closure in response to drought was non-significantly different in the two species (Student’s *t*-test: *t* = 0.22, *p* = 0.413), or in plants located at different positions in the triplet (stressed IND, directly cued T1, and relayed T2 neighbors) in either species (one-way ANOVA: *S. secundatum*: *F* = 2.13, *p* = 0.134; *C. dactylon*: *F* = 1.03, *p* = 0.367).

### 2.2. Interspecific Drought Cuing

Interplant drought cuing was observed in all interspecific treatment combinations. Subjecting one of the roots of an IND plant of either *S. secundatum* or *C. dactylon* to drought caused significant stomatal closure in both directly cued T1 and relayed cued T2 plants, regardless of species combination, but its strength varied with plant identity and position (Figure 2b,c,e,f).

Drought treatment causes similar 34–40% decreases in stomatal aperture in stressed (IND) *C. dactylon* plants and their unstressed (T1) *S. secundatum* neighbors (Figure 2e,f; non-significant difference in stomatal closure between IND and T1 plants in both C.d.–S.s.–C.d, and C.d.–S.s.–S.s. treatments; Table 1). Drought-stressed (IND) *S. secundatum* plants demonstrate 51–67% decreases in relative stomatal aperture when immediately neighboring *C. dactylon* T1 plants (Figure 2b,c; S.s.–C.d.–S.s.— Student’s *t*-test: *t =* 1.46, *p* = 0.079; S.s.–C.d.–C.d.— Student’s *t*-test: *t =* 3.00, *p* = 0.003); however, these decreases mostly resulted from increased stomatal aperture in the control S.s. IND plants when neighboring C.d. T1 plants (Figure 2b,c).

In contrast to monospecific triplet combinations (Figure 2a,d), both species demonstrate significant but weaker stomatal closure in response to relayed cuing (T2 plants) than to direct interspecific drought cuing (T1 plants), regardless of triplet combination (Figure 2b,c,e,f), with significant or marginally significant differences in stomatal closure between IND and T2 plants in all interspecific triplet combinations (Table 1).

## 3. Discussion

Adaptive phenotypic plasticity relays on the perception and integration of information pertaining to anticipated internal physiological states, growth conditions, challenges, and opportunities [8,13,15]. We previously demonstrated that certain plants can anticipatorily adapt to impending drought by perceiving root cuing from their stressed conspecific neighbors [16,35,37]. Although we cannot rule out the possibility that the observed interplant cuing involves volatile organic compounds, e.g., refs. [38,39], our previous studies demonstrated that, at least in *Pisum sativum,* interplant drought cuing is mostly, if not solely, based on inter-root cuing as it could be only observed between plants that shared their rooting media [16].

Here we demonstrate, for the first time, the existence of interspecific drought cuing and relayed cuing in plants. As expected (see [37]), both drought-stressed *S. secundatum* and *C. dactylon* plants, and their unstressed conspecific neighbors closed their stomata to similar extents (Figure 2a,d). In contrast to our expectations, we could not find a significant greater effect of drought cuing of the more xeric *C. dactylon* in comparison to its more mesic *S. secundatum* counterpart.

The greater relative stomatal width in stressed IND *S. secundatum* plants when neighboring unstressed *C. daclylon* (Figure 2b,c) could be only partially attributed to a decreased stomatal aperture in these plants but mostly to increases in stomatal aperture in the unstressed IND S. secundatum plants, suggesting a strong dependance of the responses of drought-stressed *S. secundatum* on the identity of its immediate (T1) neighbors.

As each plant is both perceiving and emitting stress cues, stress cuing and relay cuing could be expected to elicit cuing amplification by self-propagation (see [40] for a similar phenomenon related to volatile defense cuing), with an increasingly stronger response of plants to their own (echoed) cues. Such increased responses are reminiscent of amplified responsiveness of previously primed plants to later challenges such as insect herbivory [41], salt stress [42], or pathogen attack [43]. However, for such a self-amplified cuing system to be reliable, it is essential that plants do not engage in a runaway overly escalating state of alert [44]. Accordingly, it is expected that the level and effectiveness of ongoing stress cuing would strongly depend on eventual materialization of the anticipated stressful conditions, without which they are expected to rapidly habituate and drastically decrease their stress responsiveness over time [37].

Leakiness of honest cues or signals from stressed plants can be only expected where the average fitness benefits of information sharing outweigh their costs [16,37,45]. Although sharing information with potential competitors would be typically selected against, leaking drought cues could be beneficial due to the following reasons:

**Neighbor identity**: both *S. secundatum* and *C. dactylon* are clonal plants capable of creating large patches where most interactions and information sharing are between clonemates [46,47].

**Plant size and integration**: the considerable absolute size and longevity of clonal plants imply that the distance between different organs on the same clone could be substantial and the physiological integration of the clone typically deteriorates over time due to disturbances, trampling, or grazing (e.g., ref. [48]). Under such circumstances, exogenous signaling between different parts of the same clone could be more rapid and efficient than endogenous signaling [49,50].

**Facilitation**: if and to the extent that drought cuing can induce increased water use efficiency and decreased water uptake in receiver plants, drought cuing could alleviate drought and increase survival and performance of larger patches of neighboring plants, regardless of their genetic identity [45]. Such circumstances can be particularly emphasized in extreme arid environments, where the importance and prevalence of facilitation could be greater than those of competitive interactions [51,52,53].

**Diversity and stress tolerance**: the possibility that information regarding impending stresses could be exchanged between different community members may not only significantly affect the magnitude and fate of interspecific interactions, but also the ability of whole communities to tolerate or resist abiotic stresses [54,55]. Recent studies have shown that increasing species richness could enhance drought tolerance and resistance (e.g., refs. [56,57,58]. In the context of our findings, and to the extent that they are indicatory of fitness-related implications [16], the potential advantages of interspecific drought cuing could further outweigh the possible costs of sharing viable information with potential genetically alien competitors.

Our findings suggest that the effectiveness of interplant drought cuing could depend on the identities of the emitter and the receiver plants. Previous studies demonstrate that some plants are able to detect and adaptively respond according to the identity of their neighbors [59,60,61,62,63,64,65]. A recent study found that the composition of VOCs emitted from focal plants following herbivory stress was affected by the identity of their neighbors [66]. Our results are consistent with the speculation that responses to stress cues could rely on the identity of the stress cue emitters, though further work is required to study the hypothesis that responsiveness to specific stress cues could depend on the ability of the responding plants to not only perceive stress cues but also to differentially respond according to the abilities of the emitters to tolerate and resist the perceived stress.

Our findings call for further investigation into the mechanisms of intra- and interspecific stress cuing and relayed cuing, in the inherent (G), environmental (E), and interactive G X E contexts of their stress tolerance and resistance. For example, it could be expected that the ability of plants to effectively exchange stress cues and signals depends on the history of their cohabitation in the same ecosystems and geographical ranges (*sensu* [67]).

## 4. Materials and Methods

### 4.1. Plant Material

*C. dactylon* and *S. secundatum* were chosen for their ease of handling, propagation, growth, and their xeric evolutionary backgrounds. We previously demonstrated that both species are able to communicate drought cues with their conspecific neighbors [37]. *C. dactylon* (Bermuda grass) is a prostrate perennial grass, which spreads by means of both stolons and rhizomes [68]. It is common to warm ecosystems in most continents, where it occurs in diverse-types disturbed habitats and desert washes [69,70,71]. *C. dactylon* cultivars are commonly used as sturdy turf and lawn grasses [72]. *S. secundatum* (buffalo grass) is a perennial stoloniferous grass native to the Caribbean region, South America, and parts of North America and Africa, and it has been introduced to many other geographical regions [73]. *S*. *secundatum* is a strong competitor and is commonly used as a lawn grass [74]. A few studies demonstrate that *C. dactylon* is more drought-resistant than *S. secundatum* [75,76,77]. *C dactylon* was collected from natural populations near the Sede Boqer campus, Israel, and *S. secundatum* was acquired from a commercial nursery (Deshe-Itzhar, Kfar Monash, Israel) as sod.

### 4.2. Experiment Design

Testing for drought cuing required that specific stress-induced plants (IND) would experience a drought event or benign conditions while their neighboring target plants (T1, T2) would only experience cuing from the IND plants (Figure 1). This was achieved by using triplets of various combinations of split-root *S. secundatum* and *C. dactylon* plants planted in rows of four pots (Figure 1). One of the roots of the IND plant was subjected to either drought or benign conditions while its other root shared a pot with one of the roots of its nearest unstressed neighbor (T1). The other root of T1 shared its pot with one of the roots of an additional unstressed target plant (T2). This configuration permitted T1 to exchange stress cues with both IND and T2, while preventing direct root cuing between IND and T2 and, thus, allowing us to separately study the effects of direct and relayed drought cuing on T1 and T2, respectively ([35]; Figure 1a).

Drought cuing was tested in and compared between the following plant triplet combinations (Figure 1b):

Intraspecific cuing: S.s.–S.s.–S.s., C.d.–C.d.–C.d.;
Interspecific cuing (*S. secundatum* stressed-induced): S.s.–C.s.–S.s., S.s–C.d.–C.d.;
Interspecific cuing (*C. dactylon* stressed-induced): C.d–S.s.–C.d., C.d.–S.s.–S.s.

allowing us to compare both direct and relayed interplant drought cuing between intra- and interspecific neighboring configurations.

### 4.3. Growth Conditions and Experimental Setup

The plants were grown in a naturally lit greenhouse, partially controlled by an automated pad-and-fan system (Termotecnica pericoli, Albenga, Italy), under 30% sunlight at the Sede Boqer campus, Israel (30°52′ N, 34°47′ E). Plants were vegetatively propagated from 10 *C. dactylon* clones, and an unknown number of *S. secundatum* mother plants. Two-ramet cuttings were planted in moist no. 2 vermiculite and grown in the greenhouse (see above) for 14–21 d, during which each ramet regenerated 3–5 leaves and 4–6 cm long roots.

Triplets of similarly sized two-ramet plants were planted in rows of four 0.2 L, 7 cm diameter, 9 cm high pots (Miniplast, Ein Shemer, Israel). In stoloniferous plants such as *C. dactylon* and *S. secundatum,* resource translocation is commonly acropetal (e.g., ref. [78]) and in response to herbivory stress, systemic warning signals were shown to travel more rapidly acropetally than basipetally [79], implying that planting orientation might affect the rate and effectiveness of signal transmission within and among plants. To increase uniformity and the probability of finding communicative cuing, potential differential effects of axis polarity were avoided by directing the plants so their proximal ramets were rooted in (IND) or nearer (T1–T2) the induction pot (pot 1, Figure 1a).

Upon transplantation into the experimental pots, all roots were trimmed to 3 cm to encourage root regeneration and intermingling in the shared target pots. Plants were allowed to regenerate and habituate to the experimental systems for 14 days before the onset of the experiment, during which time they were individually irrigated to field capacity with 100 mL nutrient solution (Ecogan, Caesarea, Israel) every 3–4 days. Pots were individually wrapped with aluminum foil to block light from reaching the roots. Pots were individually drained into separate drip trays to prevent the seepage and capillary migration of root exudates between the pots.

To allow rapid and non-destructive initiation of drought conditions, the induction pot (pot 1, Figure 1) was filled with tap water and the other pots were filled with a commercial soil mixture (Deshanit, Be’er Yaakov, Israel).

The experiment was conducted in the greenhouse starting on April 29, 2012. Drought stress was inflicted to the proximal root of the IND plant as described in [35], by carefully pumping the water from pot 1 (orange; Figure 1a) using a flexible-tip syringe and filling it with 8 g of either dry or wet mixture of 4:1 mixture of no. 1 vermiculite (Agrekal, Habonim, Israel) and bentonite (Minerco, Netanya, Israel) (VB) for 60 min [35,37]. To account for potential handing effects, control (benign) sets were induced by filling pot 1 with a mixture of wet VB (5.5 g VB and 45 mL distilled water), reflecting the effects of drought cuing rather than potential responses to the physical handing of the plants or the chemical components of VB. Accordingly, stomatal aperture in the IND plant reflected the direct effects of partial (only to one of the two roots) drought, and stomatal aperture in the T1 and T2 plants reflected the effects of direct and relayed drought cuing, respectively.

### 4.4. Stomata Measurements

Stomatal aperture was measured for its highly sensitive responsiveness to various environmental stresses, especially drought, e.g., ref. [80]. Stomatal aperture was estimated from epidermal impressions following [35]: negative impressions of the lower surfaces of 1–2 fully unfurled 20–30 mm^2^ leaves of each sampled plant were obtained using a fresh mixture of vinyl polysiloxane dental impression silicone elastomer (Elite HD+, Badia Polesine, Rovigo, Italy). Following hardening, a positive impression of the leaf surface was obtained from the silicone molds using clear nail polish, which resulted in transparent preparations suitable for microscopic examination [35]. Stomatal aperture was estimated using AxioVision software (Carl Zeiss MicroImaging, Thornwood, NY, USA) on digital images of the nail-polish microscopic preparations. Average stomatal width was calculated from the data obtained from at least 10 stomata per plant, selected haphazardly from 2–5 0.02 mm^2^ areas in the center of each microscopic preparation. To avoid observer bias, all samples were handled and analyzed using a single-blind protocol, whereby the observer was unaware of the treatment identity of the samples.

### 4.5. Data Analyses

Stomata size greatly differed between the studied species, with *S. secundatum* having ca. double stomatal aperture than *C. dactylon* (Figure 2a,d). The main studied treatment effects were analyzed by pairwise comparisons of stomatal aperture between plant triplets in which one of the roots of the IND plant (pot 1) was treated by drought and a control triplet in which all plants were kept under well-hydrated benign conditions (Figure 1). To easily visualize and properly compare the treatment effects on the two species, we also calculated the inverse logged ratios between stomatal aperture of the treated and the control plants to provide equal weights to cases in which either the treated or the control plants in each replication pair had a larger average stomatal aperture then its counterpart [81]. Differences between treated (drought, drought cuing) and control (benign conditions) groups were tested using paired *t*-tests. Comparisons between non-paired treatment groups, such as between relative stomatal width (inv LOG ratio dry/wet; Figure 2) were carried out using either Student’s t-tests, where comparing two treatment groups or one-way ANOVAs when comparing more than two treatment groups [82]. All statistical analyses were conducted using SYSTAT 13 (SPSS).

## Figures and Tables

**Table 1:** Comparing stomatal closure between drought-treated (IND) plants and their cued (T1) and relayed cued (T2) neighbors in different interspecific triplet sets.

Triplet Combination IND–T1–T2	Difference in Relative Stomatal Width between T1 or T2 and IND	Student’s *t*-Test
	**T1 vs. IND**	
S.s.–C.d.–S.s.	+30%	*p* = 0.075
S.s–C.d.–C.d.	+78%	*p* = 0.005
C.d.–S.s.–C.d.	-6%	*p* = 0.338
C.d.–S.s.–S.s.	+19%	*p* = 0.177
	**T2 vs. IND**	
S.s.–C.d.–S.s.	+57%	*p* = 0.028
S.s–C.d.–C.d.	+100%	*p* < 0.001
C.d.–S.s.–C.d.	+24%	*p* = 0.071
C.d.–S.s.–S.s.	+28%	*p* = 0.027

## Data Availability

Not applicable.
